# Advances in vitiligo: Update on therapeutic targets

**DOI:** 10.3389/fimmu.2022.986918

**Published:** 2022-08-31

**Authors:** Yifei Feng, Yan Lu

**Affiliations:** Department of Dermatology, Jiangsu Province People’s Hospital and Nanjing Medical University First Affiliated Hospital, Nanjing, China

**Keywords:** vitiligo, targeted therapy, JAK inhibitors, biological, treatment, miRNA - microRNA, Treg

## Abstract

Vitiligo, whose treatment remains a serious concern and challenge, is an autoimmune skin disease characterized by patches of depigmentation. The increasing application of molecular-targeted therapy in skin diseases, such as psoriasis and systemic lupus erythematosus, has dramatically improved their condition. Besides, there is a favorable effect of repigmentation in the treatment of the above diseases combined with vitiligo, implying that molecular-targeted therapy may also have utility in vitiligo treatment. Recently, the role of cytokine and signaling pathways in vitiligo pathogenesis are increasingly recognized. Thus, investigations are underway targeting the molecules described above. In this paper, we present a synopsis of current practices in vitiligo treatment and introduce the improvement in identifying new molecular targets and applying molecular-targeted therapies, including those under development in vitiligo treatment, providing valuable insight into establishing further precision medicine for vitiligo patients.

## 1 Introduction

Vitiligo is a primary, circumscribed, or generalized depigmentation of the skin and mucosa, related to genetic factors, self-destruction of melanocytes, cytokines, autoimmunity, and oxidative stress (1). While the detailed molecular mechanisms still require further investigation. In recent years, various studies have showed that the IFN-γ-CXCL9/10-CXCR3 axis appears to be important in vitiligo, *via* inhibiting melanogenesis, inducing apoptosis of melanocytes, and further recruiting T cells to the skin. These are all involved in the JAK/STAT pathway. In addition, cytokine, including HSP70i, IL-15, IL-17/23, TNF as well as wnt signaling pathway, Tregs, miRNAs have also been proved to be involved in the pathogenesis of vitiligo.

Vitiligo can be treated by different modalities of phototherapy, surgical procedures, and topical therapies, such as glucocorticosteroids, immunosuppressive agents, calcineurin inhibitors, and vitamin D. However, current treatments for vitiligo remain suboptimal, which may not be equally effective in all vitiligo patients, and it would be inconvenient for patients to visit clinics for phototherapy. Targeted therapies, such as biologics targeting cytokines and small-molecule inhibitors targeting intracellular signaling molecules, are recently emerging as promising therapeutics for autoimmune diseases. Their applications also promote our understanding of the detailed molecular mechanism of vitiligo and are essential for guiding a more precise vitiligo treatment. In this article, details of the roles that related cytokines and pathways play as well as the efficacy of targeted therapy have been described.

## 2 Current treatment

Topical, systemic treatment, and phototherapy are useful for stabilization and repigmentation of vitiligo. Treatment modalities are chosen in the individual patient, based on disease severity, disease activity (stable versus progressive disease), patient preference (including cost and accessibility), and response evaluation. For rapidly progressive disease, low-dose oral glucocorticoids and phototherapy are useful in stabilizing the disease. Therapeutic options for stable, segmental vitiligo include topical therapies (eg, topical corticosteroids, topical calcineurin inhibitors), targeted phototherapy, and surgical therapy (tissue grafts and cellular grafts) (**Table 1**) (14). In recent years, attempts have been made to improve the repigmentation of vitiligo phototherapy by combination therapies, including NB-UVB with glucocorticoids (15), and topical calcineurin inhibitors (16). While their positive results were not confirmed in all studies. However, the method of treatment described, which were nonspecific, general, off-label, non-targeted with modest efficacy led to the problem of recurrence after stopping treatment. Therefore, efforts should be made to achieve a more comprehensive understanding of vitiligo pathogenesis to develop novel effective therapies (**Table 2**).

**Table 1:** Current treatment modalities for vitiligo.

Classification				Treatment	Dosage	Reference
Active				Systemic glucocorticoids	10-20mg/d	(2–4)
				Phototherapy	NBUVB	
				Systemic immunosuppressants	Cyclosporine, methotrexate, and mycophenolate mofetil	
Stable	Nonsegmental vitiligo	<10 percent of the body surface area	Localized	Topical corticosteroids	Mometasone furoate	(5)
				Topical calcineurin inhibitors	Tacrolimus (0.03% in children and 0.1% in adults) or pimecrolimus 1%	(6)
		Disseminated	Phototherapy	NBUVB	–
		Recalcitrant	Targeted phototherapy	308 nm monochromatic excimer lamps or lasers	(7)
			Psoralen plus ultraviolet A photochemotherapy	PUVA	(8)
			Transplantation procedures	Autologous suction blister grafts; Minigrafts or punch grafts, including 1 mm punch grafts; Split-thickness grafts; Laser ablation plus cultured epidermal suspensions; Autologous noncultured epidermal cell preparations, including the Jodhpur technique; Hair follicle transplantation; Autologous melanocyte cultures	(9)
		10 to 40 percent of the body surface area		Phototherapy	NBUVB	(10)
		>40 percent of the body surface area		Phototherapy	NBUVB	–
		Depigmentation	Monobenzone	(11)
	Segmental vitiligo			Topical corticosteroids		(12, 13)
				Topical calcineurin inhibitors	
				Targeted phototherapy	
				Transplantation procedures	

NBUVB, Narrow Bound Ultra Violet B.

**Table 2:** Molecular-targeted therapies for the treatment of vitiligo.

Reference	Classification	Target molecule	Agent	Study stage	Dosage	Results
(Harris et al. 2012) (17)	IFN-γ neutralizing antibody	IFN-γ	XMG-6	Mouse model/Case series	100-500 μg intraperitoneal injection twice weekly	Significantly inhibited the development of depigmentation
Richmond, Harris, Dresser, Su, Zhou, Deng, Hunter, Luster, et al. 2014) (18)	CXCL10 neutralizing antibody	CXCL10	CXCL10 neutralizing antibodies	Mouse model	100μg intraperitoneal injection 3 times weekly	Develop repigmentation after 4 weeks of treatment
(Richmond et al. 2017) (19)	CXCR3 depleting antibody	CXCR3	CXCR3 depleting antibodies	Mouse model	100μg intraperitoneal injection 3 times weekly	Reverse vitiligo in mice
(Henning et al. 2018) (20)	HSP70i encoding DNA	HSP70i	HSP70i_Q435A_ DNA delivery	Mouse model	2.5mg weekly	Develop remarkable repigmentation throughout the 6-month follow-up period
(Richmond et al. 2018) (21)	Anti-CD122 antibody	IL-15	ChMBC7	Mouse model	100 mg 3 times weekly	Significant repigmentation in treated mice
(Bhardwaj et al. 2019) (22)	Anti-IL-17 antibody	IL-17	Anti-IL-17A receptor antibody	Cell experiment	–	An increased melanin content, increased expression of TYR, MITF along with its downstream genes, and cell proliferation was observed
(Elkady et al. 2017) (23)	Anti- IL-23	IL-23	Ustekinumab	Case series	90 mg subcutaneous injection at 0 and 4 week, and subsequent every 8 weeks	The vitiligo on the face and neck was improved
(Simon and Burgos-Vargas 2008) (24)	TNF inhibitor	TNF	Infliximab	Case series	5mg/kg intravenously	Extensive pigmentation
			Etanercept	Phase 2 (NCT00134368)	Etanercept 50 mg subcutaneously once or twice weekly	Not available
(Ruiz-Arguelles et al. 2013) (25)	Anti-CD20 monoclonal antibody	CD20	Rituximab	Case series	Two 500-mg intravenous infusions	Three of five patients showed overt clinical improvement, one had slight improvement
	CTLA4-Ig	CTLA4	Abatacept	Phase 1 (NCT02281058)	self-injections of 125mg weekly	Not available
(Miao et al. 2018) (26)	PD-L1 fusion protein	PD-L1	PD-L1 fusion protein	Mouse model	–	Reversed depigmentation development in Pmel-1 vitiligo mice
	JAK inhibitor	JAK1/3	Tofacitinib	Phase 2 (NCT04246372)	5mg oral tablets BID	Not available
		JAK1/2	Ruxolitinib	Phase 2 (NCT02809976)	1.5% phosphate cream BID	4 patients presented significant facial improvement, 23% of patients decreased VASI
		JAK1/2	Baricitinib	Phase 2 (NCT04822584)	4mg/d orally	Not available
		JAK1/3	Ifidancitinib	Phase 2 (NCT03468855)	ATI-50002 topical solution 0.46% BID	Mean change in F-VASI: -0.067 (0.2411) VNS: 2.2 (0.66)
		JAK3	Ritlecitinib	Phase 2b (NCT03715829)	200 mg QD for 4 weeks followed by 50 mg QD for another 20 weeks	Mean change in F-VASI: -21.2 (4.13)
		TYK2/JAK1	Brepocitinib	Phase 2b (NCT03715829)	Not available	Not available
		SYK/JAK	Cerdulatinib	Phase 2a (NCT04103060)	0.37% Cerudulatinib gel BID	Not available
(Zou et al. 2021) (27)	Wnt-specific agonists	Wnt	SKL2001	Cell experiment	–	The expression levels of the melanogenesis-associated proteins, MITF, TYR, TRP1, and TRP2, were significantly increased

HLA, human leukocyte antigen; TCR, T cell receptor; DAMPs, damage-associated molecular patterns; TRM, resident memory T cells; JAK, Janus kinase; STAT, signal transducer and activation of transcription; CXCL, chemokine (C-X-C motif) ligand; CXCR, chemokine (C-X-C motif) receptor; TNF, tumor necrosis factor; F-VASI, Facial Vitiligo Area Scoring Index; VNS, Vitiligo Noticeability Scale.

## 3 Small molecules

### 3.1 Emerging therapeutics targeting Janus-activated kinase (JAK) signaling

The Janus kinases family consists of JAK1, JAK2, JAK3, and TYK2, which is engaged in the important JAK/STAT pathway, exhibiting pleiotropic effects on transducing multiple extracellular signals involved in regulating proliferative signaling, differentiation, migration, and apoptotic properties (28).

There are no licensed JAK/STAT inhibitors available against dermatological problems, however, some of them (ruxolitinib and tofacitinib) are used to treat other conditions such as myelofibrosis and RA. However, off-label usage of these medications in the treatment of vitiligo has shown promising outcomes.

JAK-STAT inhibitors promote Sonic Hedgehog and Wnt signaling in epidermal pigmentation, with the former inducing the migration, proliferation, and differentiation of melanocyte (29). Expanding our knowledge of these medications’ efficacy and safety profiles, as well as their use in dermatological conditions, is critical for establishing their risk-benefit ratio.

#### 3.1.1 Tofacitinib

Tofacitinib is an FDA-cleared JAK1/3 inhibitor for treating RA, PsA, and active ulcerative colitis.

Tofacitinib 5-10 mg QD/BID has demonstrated superior efficacy against vitiligo, with improvement ratios of 5.4% in 5/10 patients with sun-exposed areas or areas treated only with phototherapy (30), and a reduced rate in vitiligo area scoring index (VASI) score of 4.68 at baseline to 3.95 at 5 months in another trial (31). In addition, a decline in the number of CD8^+^ T cells and chemokines, such as CXCL9 and CXCL10 has been observed after tofacitinib treatment, but no variations were observed for the percentage of melanocyte-specific T cells (30).

Unfortunately, this oral medication is associated with a host of systemic side effects, including infections, malignancies, and cytopenia. Thus, topical JAK inhibitors may be more preferred. 11 vitiligo patients treated with 2% tofacitinib cream twice a day in conjunction with NB-UVB therapy thrice-weekly demonstrated a mean improvement of 70% in facial VASI. There was also a significant difference between facial and non-facial lesions (P=0.022) (32).

#### 3.1.2 Ruxolitinib

Ruxolitinib, the first Jakinib to get FDA approval, is a JAK1/2 inhibitor designed to deal with polycythemia vera and intermediate- and high-risk primary myelofibrosis (33).

Studies have shown that except for JAK inhibition, ruxolitinib also inhibited the differentiation and migration of DCs in vitiligo, increasing CD8^+^ cytotoxic T cell responses (34). In a double-blind phase 2 trial, 157 recruited vitiligo patients were randomized, in a 1:1:1:1:1 ratio, to receive topical ruxolitinib cream 1.5% BID, 1.5% QD, 0.5% QD, 0.15% QD, or a vehicle for 24 weeks, with the result showing considerably decreased CXCL9 and CXCL10 expression in 1.5% BID and 1.5% QD groups, and more individuals in groups receiving ruxolitinib cream 1.5% BID, 1.5% QD and 0.5% QD achieving F-VASI50, during which 1.5% BID group produced the highest responses in F-VASI50 (58%), F-VASI75 (52%), and F-VASI90 (33%). Besides, three positive responsive groups demonstrated significant repigmentation of vitiligo lesions and acceptable tolerability with a follow-up period of 52 weeks (35). Vitiligo on the face appears to respond more vigorously to therapy than non-facial lesions, reinforced by a 20-week, open-label trial in which patients with significant facial involvement experienced a 76% improvement in facial VASI scores (36). Furthermore, better repigmentation rates could be achieved both in oral and topical ruxolitinib treatment combined with phototherapy (37).

#### 3.1.3 Baricitinib

Baricitinib is a selective JAK1/2 inhibitor that inhibits signal transduction of numerous proinflammatory cytokines (38), approved for the treatment of RA. To our knowledge, there was only one case report describing repigmentation in vitiligo patients with baricitinib 4 mg daily for the treatment of RA. Besides, an ongoing phase 2 trial (NCT04822584) in which patients received a combination therapy of baricitinib 4mg/d and phototherapy is being performed.

#### 3.1.4 Ifidancitinib (ATI-50002)

Ifidancitinib is another dual JAK1/3 inhibitor for alopecia areata treatment, which is now undergoing phase II clinical trials for its application in vitiligo treatment. Patients with facial NSV(NCT03468855) receiving topical ATI-50002 BID for 24 weeks presented with an improved F-VASI and the Vitiligo Noticeability Scale (VNS) (39).

#### 3.1.5 Ritlecitinib (PF-06651600) and Brepocitinib (PF-06700841)

Ritlecitinib, an irreversible inhibitor of JAK3 and tyrosine kinase applicable to the treatment of moderate-to-severe RA (40) and Brepocitinib, a TYK2/JAK1 inhibitor, are currently undergoing evaluation of their efficacy and safety profile in active NSV in combination with phototherapy (NCT03715829) (41).

#### 3.1.6 Cerdulatinib (PRT062070)

Cerdulatinib, an SYK/JAK dual kinase inhibitor (42), has been assessed (NCT04103060) for its safety and tolerability for vitiligo treatment in topical formation (0.37% cerudulatinib gel BID).

However, additional studies are needed to determine the best-suited drug regimen and recommended dosage forms and doses to attain the optimum curative effect and minimal toxicity. As the occurrence of depigmentation after the withdrawal of JAK inhibitors, the mechanisms underlying need further exploration, and more work need to be done to corroborate the effectiveness in combination with other therapies.

### 3.2 Wnt signaling and its agonists

It has been shown that Wnt/β-catenin signaling plays a pivotal role in the proliferation, migration, and differentiation of melanocytes in vitiligo patients (29), which could be inhibited by oxidative stress (43). In addition, the Wnt/β-catenin pathway participates in the activation of MITF and its downstream enzymes (44). Intradermal injection of IWR-1 (inhibitor of Wnt response 1), a chemical inhibitor of β-catenin activation, and small interfering RNA (siRNA) against Wnt7α suppressed the number of epidermal melanocytes (45). This evidence suggested that stimulation of Wnt signaling may be an adjuvant therapy for vitiligo treatment. Micro-injury (46) as well as some phenanthridine-derived Wnt-specific agonists binding with the Axin protein have been proved to promote melanogenesis (47) and induce repigmentation.

### 3.3 Emerging therapeutics targeting microRNAs (miRNAs)

MiRNAs, which are a highly conservative small class of non-coding RNA molecules, participate in mRNA expression regulation *via* degradation or repression of mRNA translation (48). Previous studies have demonstrated that miRNAs were associated with genetic polymorphisms (e.g., miR-196a-2 rs11614913), immune response (e.g., miR-133b, miR-224-3p, miR-4712-3p, miR-3940-5p, miR-21−5p), oxidative stress (e.g., miR-135a, miR-9, miR-34a, miR-183, miR-184, miR-1, miR-25, miR-211, miR-383, miR-577, miR-421) and melanocyte functions (e.g., miR-434-5p, miR-330-5p, miR-137, miR-148, miR-145, miR-155, miR-203, miR-125, miR-377, miR-2909, miR-200c, hsa-miR-149-5p) (49–54), participating in pathological mechanism of vitiligo. These findings suggest that miRNAs may be involved in vitiligo pathogenesis *via* the modulation of vital genes expression in melanocytes and serve as novel therapeutic targets for vitiligo therapy.

There are two strategies for the therapeutic application of miRNAs: 1) anti-miRNAs, locked-nucleic acids (LNA), or antagomiRs (55) can be used to counteract the over-activation of miRNA. Short tandem target mimic (STTM)- miR-508-3p has been validated to upregulate SOX6 expression, leading to increased expression of key melanogenic genes CREB, MITF, TYR, and TYRP1/2 with increased melanogenesis (56). Besides, STTM-miR-143-5p also upregulates the expression of MYO5A, leading to an increase in the level of MITF, TYR, TYRP1, melanin, and Rab27a (57). 2) miRNA replacement, involving the reintroduction of a gene-suppressor miRNA mimic or AAV (adeno-associated virus)-mediated miRNA gain-of-function to modulate gene expression (55). A study demonstrated that the migratory capacity of melanocytes was altered by the application of miR-211 mimic through the p53-TRPM1/miR-211-MMP9 axis (58).

### 3.4 Emerging therapeutics targeting regulatory T-cells (Tregs)

Tregs are a suppressive CD4^+^ T cell subset that possesses a capacity to suppress self-reactive T cell activation and expansion (59). A clear decrease in Treg cells was observed in vitiligo skin within lesional, non-lesional, and perilesional sections (60), indicating that increasing the number of Tregs with normal function might be an important therapeutic intervention for vitiligo treatment.

Infusing purified populations of Tregs is the most direct way for the supply of Tregs. The current methods mainly include polyclonally-expanded Tregs, antigen-specific Tregs, and engineered Treg cells. In a mouse model of vitiligo, adoptive transfer of polyclonal Tregs may be effective in the short-term (61), which might however impart systemic immunosuppression (62). Besides, a TCR transgenic mouse with spontaneous vitiligo, receiving CAR Tregs treatment, developed a significant delay in depigmentation (63).

However, a limitation of infusing purified populations of Tregs might be the technical difficulty for therapeutic agent delivery to specific cells. A topical application of Tregs or the combination with CCR4 Treg homing receptor ligand CCL22 (64) by local needle-free jet injection of DNA (20) or CCL22-encoding plasmid DNA (64) may help resolve that issue. Besides, various strategies have been applied towards the modulation of Tregs function by targeting Treg-intrinsic pathways and functional modulators for Tregs. HO-1, a functional modulator of Tregs, was decreased in vitiligo Tregs. Treatment with Hemin, an agonist of HO-1, was found to enhance HO-1-induced restoration of Tregs function by up-regulating IL-10 expression (65). In addition, therapeutic method for microbiota modulation, such as neomycin treatment can significantly delay depigmentation in vitiligo mice and promote the infiltration of Tregs to the skin (66). Rapamycin, an inhibitor of PI3Kakt-mTORC1 signaling (67), efficiently halts the depigmentation process by increasing the abundance of Treg in h3TA2 mice, which effect lasted till 6 weeks after treatment (61). At present, a phase 2 clinical trial(NCT05342519) is underway for assessing the efficacy of the application of 0.1% topical rapamycin (68) (2022). In addition, nanoparticles containing rapamycin and autoantigen HEL46-61(NPHEL46-61/Rapa) were synthesized, the administration of which halted the disease progression (69). Also, the calcium-NFATc1-signaling pathway may be involved in defective Tregs function, indicating a potential therapeutic target for vitiligo treatment (70).

## 4 Cytokine-targeted therapies

Multiple monoclonal antibodies are available for vitiligo treatment, targeting IFN-γ, CXCL10, CXCR3, HSP70i, IL-15, IL-17/23, and TNF. In addition to full-size immunoglobulin, affibodies and nanobodies, composed of considerably smaller proteins, are currently being developed, which have higher bioavailability as well as affinity and specificity to the targeted molecules.

### 4.1 IFN-γ and the inhibitors

**Figure 1:**
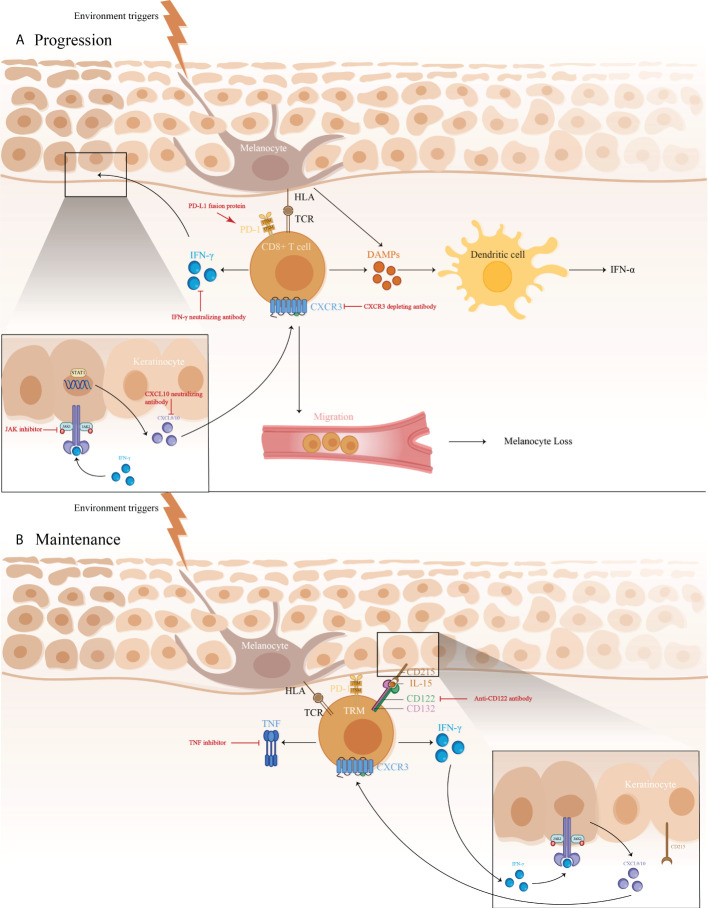
1) The immune pathogenesis of vitiligo: **(A)** CD8^+^ T cell expression of IFN-γ in vitiligo lesions activated the JAK/STAT pathway after binding to IFN-γ receptor, thus facilitating the release of CXCL9/10. The binding of CXCL9/10 to CXCR3 increased CXCR3+ T cells recruitment; **(B)** Maintenance of vitiligo lesions was influenced by the function of IL-15-dependent TRM cells, which produce IFN-γ and TNF-α. 2)Targeted therapeutic interventions in vitiligo mainly include therapies targeting IFN-γ-CXCL9/10-CXCR3 axis (IFN-γ neutralizing antibody, CXCL10 neutralizing antibody, and CXCR3 depleting antibody, as well as JAK inhibitors), anti-CD122 antibody (IL-15 receptor subunit) to decrease IFN-γ production and deplete autoreactive CD8^+^ TRM cells, TNF inhibitor to inhibit autoantibody production, and PD-L1 fusion protein to reduce the numbers of melanocyte-reactive T cells.

The IFN-γ-CXCL9/10-CXCR3 axis may be crucial for vitiligo pathogenesis, contributing to disease progression by inhibiting melanogenesis, inducing apoptosis of melanocytes, and further recruiting T cells to the skin (**Figure 1**) (71). A study showed a higher expression of IFN-γ mRNA in non-lesional and perilesional skin, especially in active vitiligo (72), which is associated with disease activity (73).

Anti-IFN-γ can have been proved to be effective in rheumatoid arthritis (RA), multiple sclerosis (MS), prevention of corneal rejection, autoimmune skin diseases, and others. In a recent study, vitiligo induction mice, treated with intraperitoneal injection with IFN-γ neutralizing antibody (XMG-6) at a dose of 100-500 μg twice a week, presented with significant improvement of depigmentation (17), with the same trend observed in vitiligo patients. Four patients who received intradermal perilesional injections presented with repigmentation of the treated area and boundary retreat (74). More research is warranted to be initiated for further definition of the role that IFN-γ plays in vitiligo and to examine whether IFN-γ neutralization would be more viable in reversing skin depigmentation.

### 4.2 CXCL10 and the inhibitors

Recent studies report a Th1/IFN-γ immune response in both human and a mouse model of vitiligo that involves elevated CXCL9, 10, and 11 productions, among which CXCL10 participated in the targeted migration of T cells (18), triggering an immune cell infiltration at the early stage (72), and involved in the downregulation of keratinocyte glycoprotein non-metastatic melanoma protein B (GPNMB) (75). A study showed that mice receiving CXCL10 neutralizing antibodies developed more repigmentation after 4 weeks’ treatment, which continued for an additional 4 weeks (18), thereby supporting CXCL10 suppression as a great therapeutic strategy.

### 4.3 CXCR3 antibodies

CXCR3 has been proved to be expressed in skin lesions, autoreactive T cells (18), and the vast majority of skin infiltrating CD8^+^ resident memory T cells (TRM), which stimulate the secretion of IFN-γ and TNF-α (76).

In a study, vitiligo mice with >75% depigmentation on their tails are treated with CXCR3 depleting antibodies for 7-8 weeks, which significantly reversed the clinical disease in a perifollicular pattern and a diminution of PMEL in the epidermis, with slightly reduced host CD8^+^ T cell numbers (19) compared to neutralizing antibody treatment (18). Although these results are preliminary, they may provide justification for further studies in targeting CXCR3 in vitiligo (19), which proposes the use of a depleting Ab to create a greater clinical efficacy by removing autoreactive cells rather than modulating their migration phenotype.

### 4.4 Inducible HSP70 (HSP70i) DNA

Indeed, HSP70i is the core participant in vitiligo predominantly through ﻿HSP70i-plasmacytoid dendritic cells (pDCs)-IFN-α-CXCL9 and CXCL10-cytotoxic T lymphocyte (CTL) axis. Pmel-1 mice vaccinated with HSP70i encoding DNA exhibited significant depigmentation, rarely seen in models knockout for HSP70i, indicating that elevated HSP70i expression alone would be enough to induce depigmentation in vitiligo prone animals (77). A study revealed that the expression of HSP-70 mRNA in skin lesions of active vitiligo patients was much higher (78), correlated with the disease activity.

Blocking HSP70i activity might have the potential to reverse vitiligo development. A recent study showed that a Sinclair swine, receiving HSP70iQ435A-encoding DNA treatment, showed remarkable repigmentation with an initial influx of T cells and increased CD4/CD8 ratios (20), which was also detected in mice with HSP70i_Q435A_-encoding DNA treatment, resulting in 76% restoration of skin pigmentation. Furthermore, the treatment halted T cells accumulation and transition to T cell phenotype in mice and human skin, engaging HSP70i_Q435A_ DNA delivery as a potent effective therapeutic intervention for vitiligo (79).

### 4.5 IL-15 and the inhibitors

It has been established that IL-15 seems to participate in IL-17 regulation and maintenance of TRM signals (80), with the latter responsible for long-term maintenance and potential relapse of vitiligo (81). The study has demonstrated a higher serum level of IL-15 in vitiligo patients than in controls, highly associated with epidermal H_2_O_2_ content and the disease activity (82, 83).

In vitiligo mice, an anti-CD122 antibody that targets IL-15 signaling was reported to effectively reverse depigmentation. Anti-CD122 therapy, either systemically or locally, decreases TRM-induced IFN-γ production and results in long-term repigmentation. These findings consider CD122-targeted drugs as a valid therapy method, which results in effective and long-lasting responses in vitiligo and other tissue-specific autoimmune disorders involving TRM (21).

### 4.6 PD-1/PD-L1 pathway

Involvement of the PD-1/PD-L1 pathway has been shown in many autoimmune diseases, including RA, MS, and vitiligo. PD-L1 expression was found limited in normal skin, and only expressed on dermal T cells, and increased in primary melanocytes and fibroblasts after exposure to IFN-γ. No such effect was seen in vitiligo patients, indicating the absence of self-protection ability for melanocytes against T-cell attack during vitiligo pathogenesis. In agreement with this, treatment with PD-L1 fusion protein reduced the numbers of melanocyte-reactive T cells, inhibited the activation of Vβ12-expressing T cells, and increased Tregs numbers, reversing depigmentation in a Pmel-1 T-cell receptor transgenic vitiligo mouse model (26). However, PD-L1 treatment may still call for extended phototherapy treatment, especially NB-UVB therapy, which likely upregulates PD-L1 expression in an NF-κB-dependent manner (84), indicating a combination use of local PD-1/PD-L1 agonistic treatment and NB-UVB therapy as a promising option.

### 4.7 Other cytokine-targeted therapies under investigation

#### 4.7.1 IL-17/23 and the inhibitors

Studies on the effect of IL-17/23 in vitiligo resulted in contradictory findings. On one hand, Th17 cells and IL-17 in vitiligo patients may inhibit function-related factors, repress melanogenesis, and dramatically induct other Th17 type cytokines as well as IL-1β production from dermal fibroblasts and keratinocytes (85). Elevated Th17 cells and IL-17/23 levels in skin lesions and serum of vitiligo patients, were positively correlated with disease activity (86, 87), and decreased after narrowband ultraviolet B (NBUVB) treatment (88). Primary melanocyte culture showed an increased expression of MITF and its downstream genes, increased melanin pigment, and cell proliferation after blockade with anti-IL-17RA (22). Besides, incidences of repigmentation have been documented in ustekinumab treatment of vitiligo (23). However, secukinumab treatment in patients with active non‐segmental vitiligo (NSV) contributed to disease progression in 7/8 patients with no general reduction in CXCL9/10, sCD25/27, Th1 cells, or cytotoxic cells, resulting in early termination of study (89). There are also reports of ustekinumab-induced new-onset vitiligo and alopecia areata. The above studies showed IL-17/23 signal may not play a direct role in vitiligo pathogenesis, which needs further investigation to confirm this conjecture.

#### 4.7.2 TNF and the inhibitors

As an anti-inflammatory mediator, TNF-α is considered to play a role in vitiligo, which may promote apoptosis in melanocytes, induce B-cell activation, increase autoantibody production, and inhibit melanogenesis (90). Recent data has shown a significantly higher expression of TNF-α in vitiligo skin. TNF inhibitors are beneficial in the treatment of plaque-type psoriasis, psoriatic arthritis (PsA), RA, and inflammatory bowel disease (IBD), arousing growing interest in their use in vitiligo.

Infliximab is a chimeric anti-TNF-α monoclonal antibody specifically binding to both soluble and membrane-bound TNF (91, 92). Intravenous infliximab is widely licensed in the treatment of RA, psoriasis, ankylosing spondylitis (AS), IBD, uveitis, and Behcet’s disease. A 24-year-old patient with ankylosing spondylitis and refractory vitiligo improved significantly following six months of infliximab therapy at a dose of 5mg/kg intravenously in weeks 0, 2, and 6, and then every eight weeks for ten months (24). Besides, Etanercept is a monoclonal antibody targeted against TNF-α (93), which has been approved for the treatment of RA, juvenile RA, AS, psoriasis, and PsA. Treatment with etanercept 50 mg subcutaneously once or twice weekly for at least 2 months has shown a great curative effect on established vitiligo (94).

However, it has been shown that anti‐TNF‐α agents, especially adalimumab and infliximab (95), may exacerbate established vitiligo and induce new-onset vitiligo during treatment of other autoimmune diseases, including AS (96), Crohn’s disease (97), ulcerative colitis (98), psoriasis (99), and RA (100). The mechanism responsible for the TNF-α inhibitors-induced vitiligo is not fully understood. On the one hand, TNF-α inhibitors may increase the nucleosome-mediated autoantibody formation, interfere with the cytotoxic T-cell suppression of autoreactive B cells, and decrease Treg synthesis and activation. Additionally, infliximab increases pDC-produced IFN-γ, participating in further T cells recruiting. Although very rare, new-onset or exacerbations of vitiligo can occur in the anti‐TNF‐α treatment of other autoimmune diseases, the risk of which must not be ignored.

#### 4.7.3 Rituximab

Rituximab has specific affinity for the B-lymphocyte transmembrane protein, CD20, which is expressed on B cells (101), participating in the activation of the CD8^+^ T cells and the ensuing autoreactive reaction (102). Rituximab is licensed for the treatment of lymphomas, leukemias, transplant rejection crisis, and a series of autoimmune diseases (103, 104). An intravenous infusion of Rituximab was administered to five active disseminated vitiligo patients, the three of whom exhibited a considerable improvement in both the disease’s symptoms and histology (25).

#### 4.7.4 Abatacept

Abatacept, a fusion protein consisting of IgG1 coupled to the extracellular domain of CTLA-4 *via* the immunoglobulin’s Fc region, was licensed for treating moderate to severe RA. Ten eligible patients with active vitiligo have been included to receive self-injections of 125mg abatacept weekly from week 0 to week 24. Secondary endpoints will be evaluated during a 32-week follow-up visit (105).

## 5 Future therapeutic prospects

As a future direction, new therapeutic approaches should be developed to reduce vitiligo progression. Among the new approaches being developed, the strategy of targeting the IFN-γ-CXCL9/10-CXCR3 axis has been clinically tested. OPZELURA has been indicated for the topical treatment of nonsegmental vitiligo in adult and pediatric patients 12 years of age and older. MiRNA-based therapeutics are also in development. However, the absence of organ or tissue selectivity may also lead to off-target side effects, which must be considered and excluded in the process of miRNA-based therapeutics development. Besides, a suitable vector system, as well as the assurance of chemical and biological stability should also be taken into account. Adoptive Treg cell therapy has also been the research hotspot in recent years. However, it has always been a difficult point for reassurance for safety and the development of the delivery system.

Treating vitiligo remains a challenge. As is presented in this paper, a greater variety of precision treatments is currently being studied. With a better understanding and further validation of these therapeutic targets, patients can be stratified to achieve individualized treatment.

## 6 Conclusion

Current models of treatment for vitiligo are often nonspecific and general. Various therapy options are available for active vitiligo patients, including systemic glucocorticoids, phototherapy, and systemic immunosuppressants. While stable vitiligo patients may benefit from topical corticosteroids, topical calcineurin inhibitors, phototherapy, as well as transplantation procedures. Recently, a better understanding of the pathophysiological processes of vitiligo led to the advent of novel targeted therapies. To date, JAK inhibitors are the only category that has been proved to have a good tolerability profile and functional outcomes in vitiligo treatment, even though the risk of activation of latent infection and systemic side effects still existed, like other immunosuppressive agents. Research is in progress to investigate the important cytokines involved in the pathogenesis of vitiligo, including IFN-γ, CXCL10, CXCR3, HSP70i, IL-15, IL-17/23, and TNF, the blockade of which has undergone preliminary attempts in animal models and some patients. In addition, studies on miRNA-based therapeutics as well as adoptive Treg cell therapy are still primary, and more studies are necessary.

## Author contributions

YFF and YL contributed to the conceptual design, writing, editing, and generation of figures for this manuscript. All authors contributed to the article and approved the submitted version.

## Conflict of interest

The authors declare that the research was conducted in the absence of any commercial or financial relationships that could be construed as a potential conflict of interest.

## Publisher’s note

All claims expressed in this article are solely those of the authors and do not necessarily represent those of their affiliated organizations, or those of the publisher, the editors and the reviewers. Any product that may be evaluated in this article, or claim that may be made by its manufacturer, is not guaranteed or endorsed by the publisher.
